# LPS-Induced G-CSF Expression in Macrophages Is Mediated by ERK2, but Not ERK1

**DOI:** 10.1371/journal.pone.0129685

**Published:** 2015-06-26

**Authors:** Shwu-Fen Chang, Shih-Shan Lin, Hui-Ching Yang, Yuan-Yi Chou, Jhen-I Gao, Shao-Chun Lu

**Affiliations:** 1 Graduate Institute of Medical Sciences, School of Medicine, Taipei Medical University, Taipei, Taiwan; 2 Department of Biochemistry and Molecular Biology, College of Medicine, National Taiwan University, Taipei, Taiwan; China Medical University, TAIWAN

## Abstract

Granulocyte colony-stimulating factor (G-CSF) selectively stimulates proliferation and differentiation of neutrophil progenitors which play important roles in host defense against infectious agents. However, persistent G-CSF production often leads to neutrophilia and excessive inflammatory reactions. There is therefore a need to understand the mechanism regulating G-CSF expression. In this study, we showed that U0126, a MEK1/2 inhibitor, decreases lipopolysaccharide (LPS)-stimulated G-CSF promoter activity, mRNA expression and protein secretion. Using short hairpin RNA knockdown, we demonstrated that ERK2, and not ERK1, involves in LPS-induced G-CSF expression, but not LPS-regulated expression of TNF-α. Reporter assays showed that ERK2 and C/EBPβ synergistically activate G-CSF promoter activity. Further chromatin immunoprecipitation (ChIP) assays revealed that U0126 inhibits LPS-induced binding of NF-κB (p50/p65) and C/EBPβ to the G-CSF promoter, but not their nuclear protein levels. Knockdown of ERK2 inhibits LPS-induced accessibility of the G-CSF promoter region to DNase I, suggesting that chromatin remodeling may occur. These findings clarify that ERK2, rather than ERK1, mediates LPS-induced G-CSF expression in macrophages by remodeling chromatin, and stimulates C/EBPβ-dependent activation of the G-CSF promoter. This study provides a potential target for regulating G-CSF expression.

## Introduction

Granulocyte colony-stimulating factor (G-CSF), a hematopoietic growth factor, regulates the proliferation of neutrophil progenitors, and the differentiation of granulocyte lineages, and the survival and maturation of neutrophil progenitors, and their mobilization from bone marrow to peripheral tissues [1]. For decades, recombinant G-CSF has been widely used in patients receiving chemotherapy to increase the number of circulating hematopoietic progenitor cells and in certain patients with neutropenia. Endogenous G-CSF is produced by various types of cells, including bone marrow stromal cells, endothelial cells, macrophages, and fibroblasts, and its production is induced by inflammatory stimuli, including cytokines, such as IL-1β and TNF-α, and pathogenic toxins, such as lipopolysaccharide (LPS), via transcriptional and post-transcriptional mechanisms [2, 3]. NF-κB, NF-IL6 (C/EBP-β), and octamer-binding factor 2 (Oct-2), are transcription factors that have been identified essential for LPS-induced G-CSF expression in macrophages [4–6], but none of these factors alone is sufficient to drive LPS’s effect on G-CSF expression. Post-transcriptionally, LPS or cytokines increases G-CSF mRNA stability, which is regulated by the AU-destabilizing element and stem-loop destabilizing element in the 3’-end untranslated region [7, 8].

G-CSF stimulates the proliferation and functional maturation of neutrophils and plays an important role in host defense against microbial infection. However, excessive G-CSF levels are associated with increased severity of inflammatory diseases, for example, acute lung injury and rheumatoid arthritis [9, 10]. This is primarily due to G-CSF-induced neutrophil infiltration into the inflamed tissue and increased production of inflammatory mediators, such as cytokines, chemokines, and serum complement, which subsequently amplify the local inflammatory response. G-CSF has therefore been suggested as a molecular target for chronic inflammatory diseases [10–12]. Several studies have reported that G-CSF can also be produced by non-hematopoietic malignant tumors, such as hepatocellular carcinoma, pancreatic cancer, lung cancer, and gastric cancer, or cell lines derived from these [13–16]. G-CSF-producing tumors are often associated with aggressive growth and patients with this type of tumor tend to have a poor prognosis [16]. However, little is known about the pathological significance of G-CSF production by tumors and the underlying mechanisms triggering G-CSF expression.

It is known that LPS activates the NF-κB pathway and all three MAPK pathways (ERK, JNK/SAPK, and p38α), leading to a wide range of cellular responses, including cell differentiation, survival or apoptosis, and inflammatory responses [17]. We have previously reported that pretreatment with rapamycin, which blocks the activity of mTOR complex 1 (mTORC1), inhibits LPS-induced G-CSF expression by decreasing the expression of Oct-2, a crucial transcription factor required for this process [6]. In addition, our preliminary data showed that pretreatment for 30 min with 10 μM U0126, a specific MAP/ERK kinase inhibitor, inhibited LPS-induced expression of G-CSF in RAW264.7 murine macrophage cells (S1 Fig). In monocytes/macrophages, both extracellular signaling-regulated kinases, ERK1 and ERK2, are activated by LPS or cytokines, increasing proinflammatory gene expression [18, 19]. In response to stimuli, ERKs are phosphorylated at the Thr-Glu-Tyr (TEY) motif, and then activate numerous downstream modulators, including transcription factors Elk-1, NF-AT, STAT3, and C/EBPβ [20–22]. However, little is known about the specific involvement of ERK1 or ERK2 in LPS-induced G-CSF expression. We recently reported that ERK2 is important in G-CSF production of cancer cells [23]. In the present study, we investigated the role of ERKs in LPS-induced G-CSF expression in macrophages and identified the essential role of ERK2 in this process. Our results demonstrated that LPS-activated ERK2 functions by remodeling local chromatin, interacting with C/EBPβ and synergizing its transactivation activity to increase G-CSF expression. This study suggests that ERK2 may be a critical therapeutic target for surplus G-CSF related diseases.

## Materials and Methods

### Materials

Dulbecco’s modified Eagle’s medium (DMEM) and fetal bovine serum (FBS) were obtained from Hyclone Laboratories (Logan, UT, USA). LPS from *Escherichia coli* (serotype 0111:B4) was purchased from Sigma-Aldrich (St. Louis, MO, USA) and dissolved as a 1 mg/mL stock solution in PBS. MMLV reverse transcriptase was from Promega (Madison, WI, USA). SuperFect Transfection reagent was purchased from Qiagen (Hilden, Germany). Antibodies against ERK, phospho-ERK, p50, p65, C/EBPβ, β-actin, or lamin B were obtained from Santa Cruz Biotechnology (Santa Cruz, CA, USA). U0126 and PD98059 (specific MEK inhibitors) were purchased from Calbiochem (San Diego, CA) and were dissolved as stock solutions in DMSO, and were added to the culture medium 30 min before other treatments or as indicated with 0.1% DMSO in culture medium as the control.

### Cell culture and LPS treatment

The RAW264.7 murine macrophage cell line was cultured as described previously [6]. THP-1, a human acute monocytic leukemia cell line, was maintained and induced to differentiate into macrophages using 160 nM of phorbol 12-myristate-13-acetate (PMA) as described previously [24]. G-CSF mRNA and protein levels were compared in untreated cells and cells pretreated with the indicated concentration of inhibitor or DMSO for 30 min, then LPS was added and the cells incubated for the indicated time; unless otherwise stated, LPS treatment was at 100 ng/ml for 6 h and the concentration of U0126 was 10 μM.

### RNA isolation and RT-PCR analyses

Total cellular RNA was isolated from cells using TRIzol reagent (Invitrogen) according to the manufacturer’s protocol and its concentration was determined from the absorbance at 260 nm, then it was subjected to reverse transcription (RT)-polymerase chain reaction (PCR) analysis as described previously [6]. G-CSF, TNF-α, and GAPDH mRNA levels were determined by RT-PCR using the primers listed in S1 Table.

### Preparation of cytosolic and nuclear extracts

Cytosolic and nuclear extracts were prepared as described previously [25]. Briefly, cells were washed twice with ice-cold PBS, incubated on ice for 15 min with lysis buffer (10 mM HEPES, pH 7.4, 10 mM NaCl, 1.5 mM MgCl_2_, 0.5 mM DTT, 0.2% Nonidet P-40, and 0.2 mM PMSF), and collected by gentle pipetting. After centrifugation at 500 xg for 5 min at 4°C, the supernatant was collected as the cytosolic extract, and the pellet was extracted by incubation for 15 min at 4°C with intermittent vortexing in nuclear extract buffer (20 mM HEPES-KOH, pH 7.9, 25% glycerol, 420 mM NaCl, 1.5 mM MgCl_2_, 0.2 mM EDTA, 0.5 mM DTT, 0.2 mM PMSF, and 1x protease inhibitor cocktail), followed by centrifugation at 12,900 xg for 10 min at 4°C, after which the supernatant was collected as the nuclear extract. The Bradford method (DC Protein Assay; Bio-Rad) was used to measure the protein concentrations in the extracts, which were then stored in aliquots at—80°C.

### Western blot analysis

Cells were washed with ice-cold PBS, then lysed with RIPA buffer (20 mM Tris-HCl, pH 7.5, 150 mM NaCl, 5 mM EDTA, 0.5% NP-40, 0.5% Triton X-100, 0.1% SDS, 1 mM NaF, 1 mM PMSF, 1 mM Na_3_VO_4_, and 1 μg/ml of leupeptin), then samples of the cell lysate (20 μg of protein/lane) were separated by 10% SDS-PAGE and transferred to a PVDF membrane, which was then blocked overnight at 4ºC with blocking buffer (10 mM Tris-HCl, pH 8.0, 150 mM NaCl, 0.1% Tween 20, and 5% fat-free milk). The blots were then incubated for 1 h at room temperature with 0.5 μg/ml of rabbit polyclonal antibody against p50, p65, ERK, phospho-ERK, or β-actin in blocking buffer, then for 40 min at room temperature with peroxidase-conjugated anti-rabbit IgG antibodies (Amersham-Pharmacia Biotech) in blocking buffer. Bound antibody was detected using an improved chemiluminescence detection system (NEN Life Science Products). Protein concentrations were determined by the Bradford method (DC Protein Assay, Bio-Rad).

### Quantitation of G-CSF in culture medium

The concentrations of human (THP-1 cells) or mouse (RAW264.7 cells) G-CSF in the culture medium were measured by enzyme-linked immunosorbent assay using human or mouse G-CSF Quantikine ELISA kits (R&D Systems) according to the manufacturer's instructions.

### Plasmids

Expression plasmids encoding mouse p50, Oct-2, or C/EBPβ were, respectively, generous gifts from Dr. Neil D. Perkins, University of Michigan, Dr. Winship Herr, Cold Spring Harbor Laboratory, and Dr. Gerard Elbery, Section of Nephrology, Department of Pediatrics, University of Oklahoma Health Sciences Center. Plasmids encoding the T188A or S64A mutant of C/EBPβ were generated from the wild-type C/EBPβ-expressing plasmid by PCR mutagenesis and confirmed by sequencing analysis. A plasmid expressing the constitutively active form of ERK2 (CA-ERK2) was obtained from Upstate Co. (Charlottesville, VA, USA). All plasmids were amplified in the *E*. *coli* DH5α host strain and purified by equilibrium centrifugation in a CsCl−EtBr gradient as described previously [24]. The purity and stability of plasmid preparations were confirmed by agarose gel electrophoresis followed by ethidium bromide staining, and the DNA concentration was measured by UV absorption at 260 nm.

### Transient transfection and reporter gene activity assay

Transient transfection was carried out as described previously [25]. Briefly, RAW264.7 cells were plated and cultured in 24-well plates overnight before transfection. To determine the role of ERK in regulating the G-CSF promoter activity, 0.5 μg of the pG-CSF(-283/+35)-Luc plasmid and 0.1 μg of the phRLTK plasmid were mixed with 0.15–0.6 μg of the expression plasmid containing p50, Oct-2, CA-ERK, or C/EBPβ cDNA or mixtures of the plasmid encoding C/EBPβ and that encoding p50, Oct-2, or CA-ERK, and the total amount of DNA was adjusted to 1.2 μg with pcDNA3.1. The DNA mixture was transfected into RAW264.7 cells using SuperFect transfection reagent, then 24 h later, cell lysates were prepared using the lysis buffer in the kit and *Photinus* and *Renilla* luciferase activities were measured using the Dual-Luciferase reporter assay system as described previously [25]. The light intensity produced by *Photinus* luciferase (test plasmid) was normalized to that produced by *Renilla* luciferase (control plasmid).

### Knockdown of ERK1 and ERK2 by RNA interference

pLKO.1-shRNA plasmids encoding shRNAs targeting firefly luciferase, human ERK1 (shMAPK3-B1/ERK1i-a: 5′–CTATACCAAGTCCATCGACAT–3′ or MAPK3-A2/ERK1i-b: 5′–CAACATGAAGGCCCGAAACTA–3′), or human ERK2 (shMAPK1-F1/ERK2i-a: 5′–CAAAGTTCGAGTAGCTATCAA–3′ or shMAPK1-G1/ERK2i-b: TATCCATTCAGCTAACGTTCT) were obtained from the National RNAi Core Facility of the Academia Sinica, Taiwan. These plasmids were transfected, together with pMD.G and pCMV delta8.91, into a HEK293T packaging cell line using the calcium phosphate method and virus supernatants were collected from the medium 60 h after transfection [23]. For knockdown experiments, THP-1 cells were transduced for 24 h with the collected virus supernatants plus polybrene (8 μg/ml) and infected cells were selected with puromycin (10 μg/ml) for 10 days.

### Chromatin immunoprecipitation (ChIP) assay

The ChIP assay was performed according to the manufacturer’s instructions (Upstate Biotechnology Inc.) as described previously [6]. Briefly, after treatment, the cells were fixed with 1% formaldehyde for 10 min at 37°C to cross-link DNA and protein, collected by scraping, and sonicated to fragment the chromatin. Immunoprecipitation was performed using control rabbit IgG or rabbit antibodies against ERK, C/EBPβ, p50, p65, or histone 3 (Santa Cruz), the cross-links were reversed at 65°C for 4 h and digested with proteinase K (Sigma-Aldrich) for 1 h at 45°C to remove proteins, then the immunoprecipitated DNA was recovered by phenol/chloroform extraction and ethanol precipitation before being used as template for PCR with the following primers: G-CSF promoter (-248 to -70), forward (5'–TGGCTGGaagAGAGGAAGAG–3') and reverse (5'–CTGGGGCAACTCAGGCTTA–3') or TNF-α promoter (-270 to -4), forward (5'–CTGATTGGCCCCAGATTG–3') and reverse (5'–CTTCTGCTGGCTGGCTGT–3') (GenBank: Y00467.1). Ten percent of the chromatin DNA used for immunoprecipitation was also subjected to PCR analysis (indicated as “input”). The number of PCR cycles was 30 or 31 for all ChIP experiments and 24 for the input samples.

### DNase I accessibility assay

After 1% formaldehyde treatment, the cells were gently lysed with 500 μl of ice-cold Nonidet P-40 lysis buffer (10 mM Tris-HCl, pH 7.4, 10 mM NaCl, 3 mM MgCl_2_, 0.5% Nonidet P-40, 0.15 mM spermine, 0.5 mM spermidine), and the lysates centrifuged at 500 xg for 5 min at 4°C to pellet the nuclei, which were washed with 200 μl of Nonidet P-40 lysis buffer and centrifuged again at 500 xg for 5 min at 4°C. After gentle rinsing with 200 μl of ice-cold buffer A (100 mM NaCl, 50 mM Tris-HCl, pH 8.0, 3 mM MgCl_2_, 0.15 mM spermine, and 0.5 mM spermidine), the pelleted nuclei were suspended in buffer A containing 1 μM CaCl_2_ for DNase I (Promega) treatment, which was performed at 37°C for 5 min for THP-1 cells or for 2 min for Raw264.7 cells, and stopped with ice-cold 10 mM EDTA, pH 8.0. After reversal of the formaldehyde cross-linking at 65°C for 4 h, proteinase K and RNase A were added to the sample, which were then incubated at 37°C overnight, then DNA was extracted using the standard phenol-chloroform technique. Care was taken to use cut-off tips and very gentle pipetting to reduce non-specific DNA sheering. After precipitation, the DNA was dissolved in H_2_O.

Real-time PCR was used to identify the DNase-sensitive sites using the primers (final concentration 400 nM) listed in S1 Table. DNase I-treated and non-digested DNA were tested in triplicate. All PCR reactions were performed with SYBR Green PCR Master Mix in an ABI PRISM 7300 Sequence Detection System (Applied Biosystems 7300 System Sequence Detection System software version 1.3.) ΔCt values were determined by subtracting the Ct value of the control reaction. The conditions for the PCR were as follows: 50°C for 2 min, 95°C for 10 min, 40 cycles of 95°C for 15 sec, and 60°C for 1 min. At the end of the PCR cycles, amplification specificity was confirmed by dissociation curve analysis, and the products were separated on a 3% agarose gel and stained with ethidium bromide for visual confirmation of the PCR product.

### Statistical analyses

Results are shown as the mean + SD. Differences between mean values were evaluated using Student’s t test and were considered significant at p <0.05.

## Results

### Inhibition of ERK activation prevents LPS-induced G-CSF expression in macrophages

**Fig 1:**
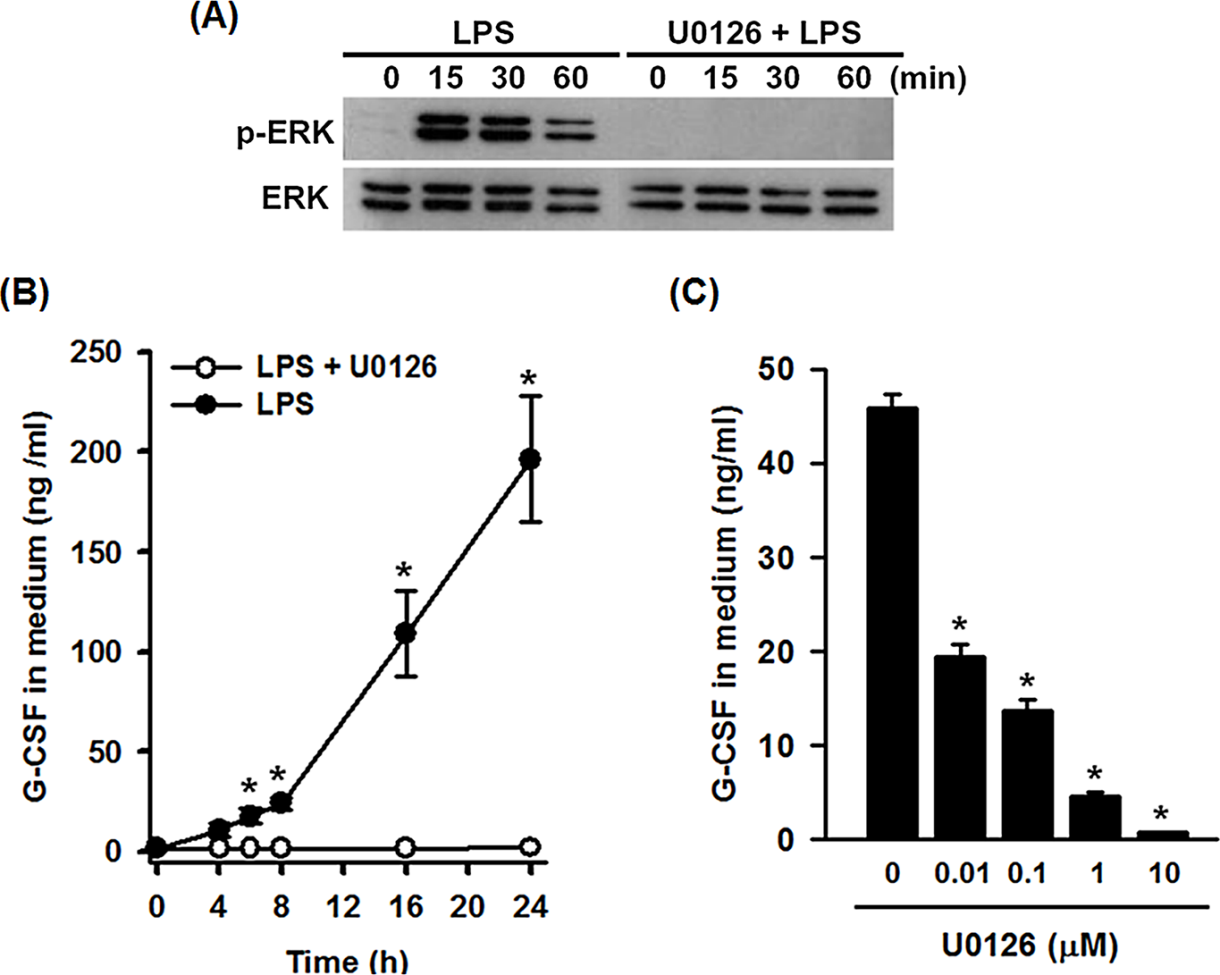
U0126 inhibits ERK1/2 phosphorylation and G-CSF expression in LPS-stimulated RAW264.7 macrophages. (**A**) Cells were left untreated (lane 1) or were incubated either with LPS (100 ng/ml) for 15 to 60 min (lanes 2–4) or with DMSO or U0126 (10 μM) for 30 min, followed by addition of same concentration of LPS and incubation for 0 to 60 min (lanes 5–8), then phosphorylated ERK1/2 and total ERK1/2 were analyzed by Western blotting. The results shown are representative of those obtained in three separate experiments. (**B**) Cells were incubated with DMSO or U0126 (10 μM) for 30 minutes, then with LPS (100 ng/ml) for the indicated time, then G-CSF levels in the medium were measured by ELISA. (**C**) Cells were incubated with DMSO or 0.01, 0.1, 1, or 10 μM of U0126 for 30 minutes, then with LPS (100 ng/ml) for 6 h and G-CSF protein levels in the medium were measured by ELISA. The results in B and C are the mean ± SD for three independent experiments (*p<0.01).

An initial experiment was designed to confirm the involvement of ERK activation in LPS-stimulated G-CSF production in macrophages. Phosphorylation of ERK1/2 was detected in mouse Raw264.7 macrophages after 15 to 60 min of treatment with LPS (100 ng/ml) and was blocked by 30 min pretreatment with U0126 (10 μM) (Fig 1A). To examine whether U0126 inhibited LPS-induced G-CSF production, Raw264.7 cells were incubated with or without U0126, then with LPS for 0 to 24 h. Fig 1B shows that LPS-induced G-CSF secretion in the culture medium was time-dependent and was abolished by U0126 pretreatment. In addition, the inhibitory effect of U0126 on LPS-induced G-CSF production was dose-dependent (Fig 1C). Since LPS is known to increase G-CSF mRNA expression in mouse bone marrow-derived macrophage (BMDMs), we then examined whether the ERK inhibitors U0126 or PD98059 could block this effect in primary cells. The results in S2A and S2B Fig show that G-CSF mRNA levels were significantly increased after LPS treatment for 6 h and this effect was blocked by pretreatment cells with 0.01 or 0.1 μM of U0126 or 1 or 10 μM of PD98059.

### ERK2 is critical for LPS-induced G-CSF expression

**Fig 2:**
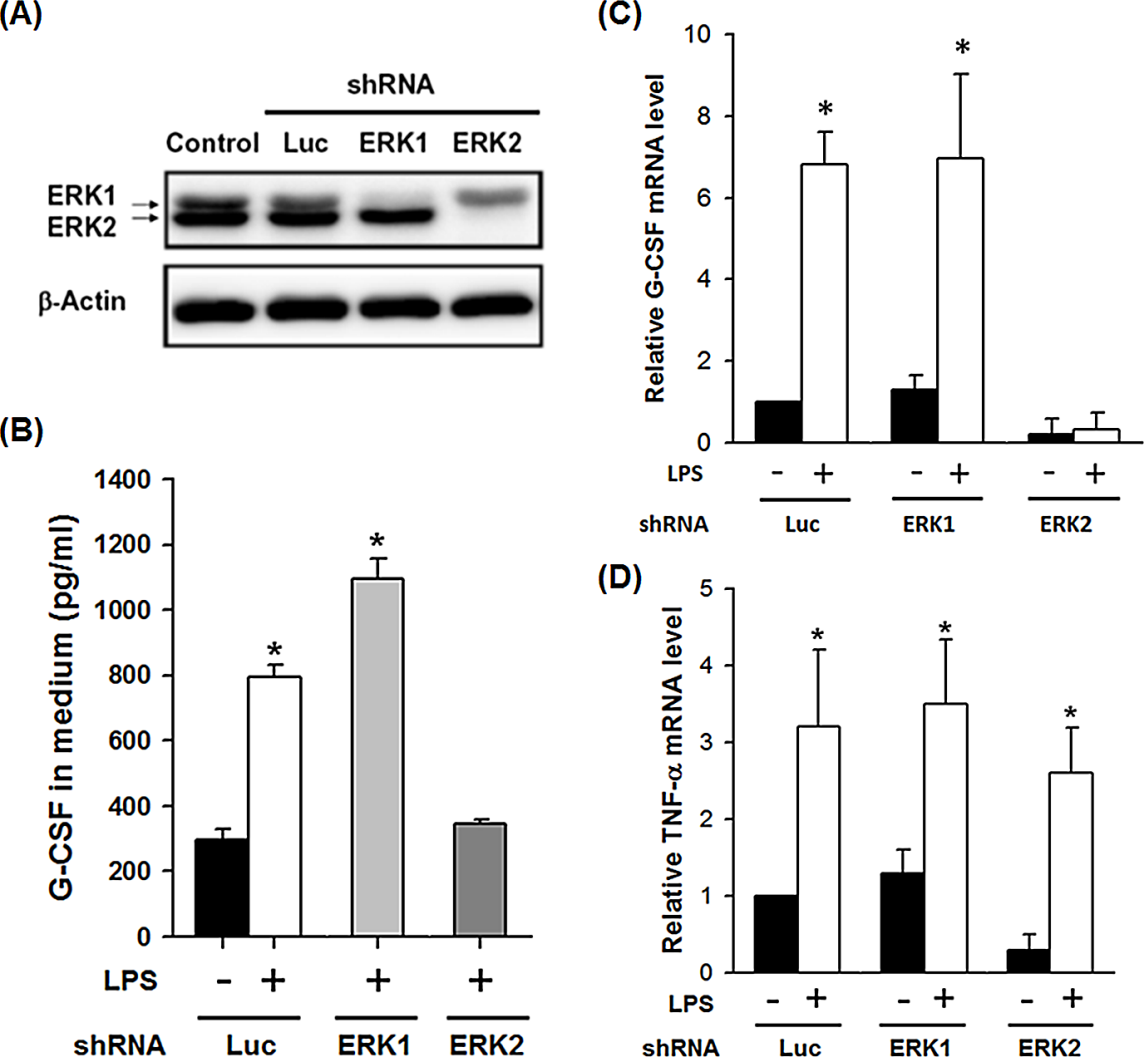
ERK2 knockdown in THP-1 macrophages blocks LPS-induced G-CSF expression. THP-1 cells were infected with lentivirus carrying specific shRNA for ERK1 or ERK2 and induced to differentiate by incubation with PMA (160 nM) for 3 days; lentivirus carrying luciferase shRNA (Luc) was used as a control, then the following tests were performed. (**A**) Levels of ERK1/2 and β-actin in the cells were determined by Western blotting; the data shown are typical of the results of three experiments. (**B**) Cells were treated with LPS (100 ng/ml) for 8 h, then G-CSF levels in the medium were determined by ELISA; untreated Luc cells were used as the control. (**C** and **D**) Levels of G-CSF mRNA (**C**) and TNF-α mRNA (**D**) in cells were determined by RT-qPCR, normalized to the levels of GAPDH mRNA, and expressed relative to levels in the Luc control (relative value = 1). In (B-D), the results are the mean ± SD for five independent experiments. *p<0.05 compared to the corresponding cells not treated with LPS.

ERK1 and ERK2 are isoforms of the ‘classical’ MAPKs and both are activated by MAP/ERK kinase 1 (MEK1) and MEK2, members of the MAPKK family. To examine whether U0126 inhibited LPS-induced phosphorylation of ERK1/2 in THP-1 macrophages, cells were induced to differentiate into macrophages by incubation with 160 nM PMA, and then were incubated with LPS for 0.5, 1 or 2 h. Phosphorylation of ERK1/2 was detected in THP-1 macrophages after treatment with LPS (100 ng/ml) and was blocked by 30 min pretreatment with U0126 (10 μM) (S3 Fig). To determine whether ERK1 and/or ERK2 involved in LPS-induced G-CSF expression, ERK expression in THP-1 human acute monocytic leukemia cells was knocked down using shRNA clones ERK1a and ERK1b carrying two different ERK1 sequences or shRNAs ERK2a and ERK2b carrying two different ERK2 sequences. The luciferase shRNA was used as a negative control. Knockdown efficiency and specificity were confirmed by Western blotting analysis (Fig 2A and S4 Fig). After shRNA-lentiviral transduction and puromycin selection, the cells were induced to differentiate into macrophages by incubation with 160 nM PMA, and then were incubated with LPS or PBS for 4 h. As shown in Fig 2B and 2C, a significant increase at LPS-induced G-CSF protein and mRNA levels was detected in control luciferase shRNA knockdown and ERK1 knockdown cells, but not in ERK2 knockdown cells. Whereas, neither ERK1 nor ERK2 knockdown had a significant effect on LPS-induced TNF-α mRNA expression (Fig 2D), though both basal and LPS-induced TNF-α mRNA levels were slightly lower in the ERK2 knockdown cells. These results show that ERK2, but not ERK1, is essential for LPS-induced G-CSF expression, but is not a common regulator for the expression of LPS-activated genes.

### Cooperative regulation of G-CSF promoter activation by ERK2 and C/EBPβ

**Fig 3:**
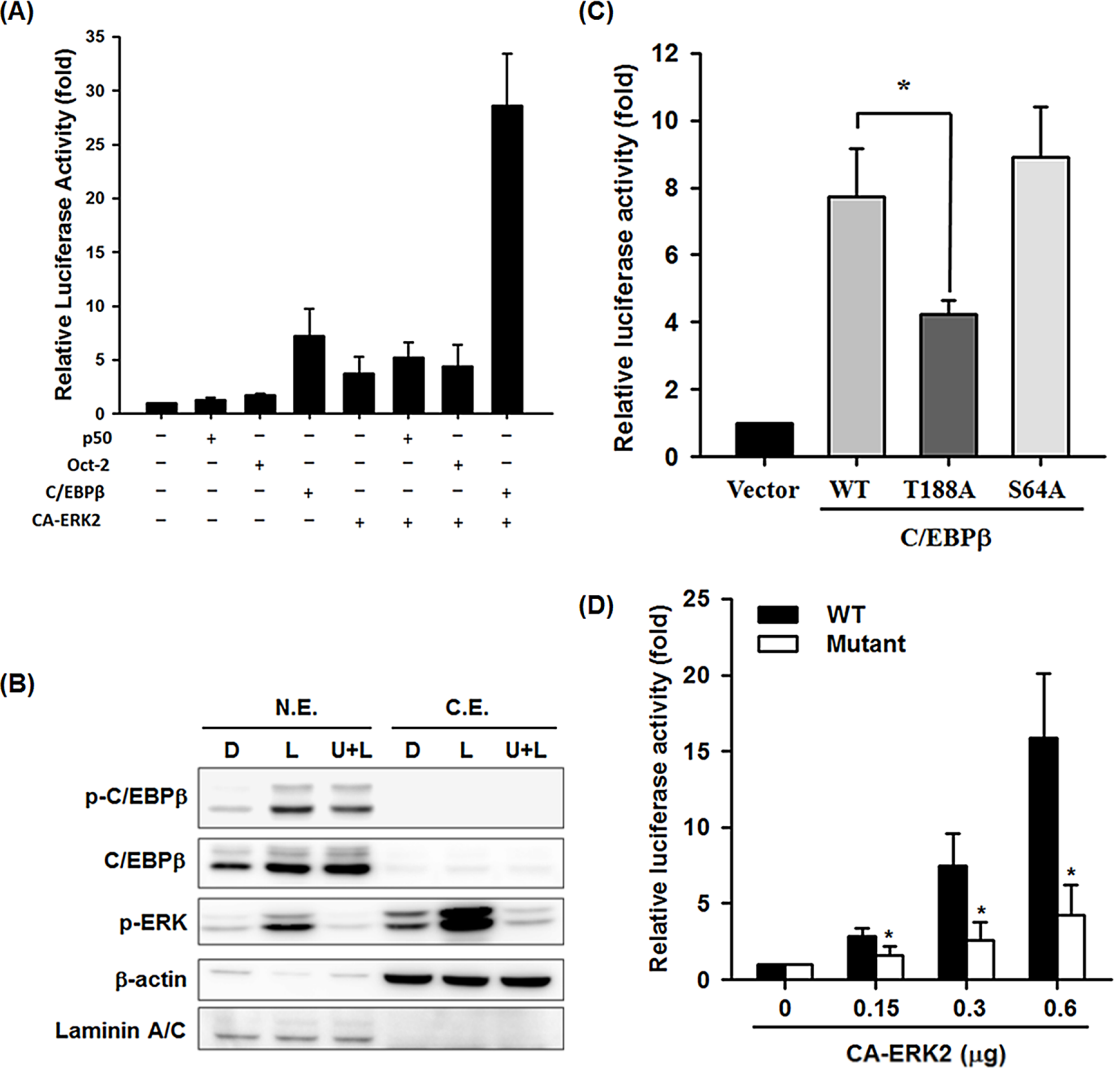
Threonine188 of C/EBPβ is important for the interaction of ERK2 with C/EBPβ that activates G-CSF promoter. (**A**) RAW264.7 cells were co-transfected with a mixture of two reporter plasmids (pG-CSF(-283/+35)-Luc and phRLTK) and the CA-ERK2, p50, Oct-2, or C/EBPβ plasmid alone or the C/EBPβ plasmid plus the CA-ERK2, p50, or Oct-2 plasmid, then luciferase activity was measured at 24 h after transfection using the Dual-luciferase reporter assay system. (**B**) RAW264.7 cells were pretreated for 30 min with DMSO (D) or U0126 (U) (10 μM), then were incubated with LPS (100 ng/ml) (L) for another 4 h, after which a nuclear extract (N.E.) and cytosolic extract (C.E.) were isolated and levels of total and phosphorylated C/EBPβ, phosphorylated ERK1/2, β-actin (cytosol internal control), and laminin A/C (nuclear internal control) analyzed by Western blotting. The data shown are typical of those obtained in three experiments. (**C**) Cells were cotransfected with reporter plasmids (0.5 μg of pG-CSF (-283/+35)-Luc mixed with 0.1 μg of phRLTK), 0.15 μg of CA-ERK expression plasmid, and 0.15 μg of the WT, T188A, or S64A C/EBP expression plasmid or 0.15 μg of pcDNA3.1 (vector control), then the reporter luciferase activities were analyzed 24 h after transfection. (**D**) Cells were co-transfected with pG-CSF(-283/+35)-Luc carrying the wild type or mutant C/EBPβ binding site, 0, 0.15, 0.3, 0.6 μg of CA-ERK2 expression plasmid, and the internal control phRLTK, then luciferase activities were measured 24 h after transfection. All luciferase activities are shown relative to the vector control (relative value = 1). In A, C, and D, values are the mean ± SD for three independent experiments. *p<0.05 compared to wild-type cells.

The results above suggested that ERK2 might involve in LPS-activated G-CSF expression at the transcriptional level. To further clarify whether ERK2 activation affected the G-CSF promoter activity, reporter assays were performed. Raw264.7 cells were transfected with a reporter plasmid containing the luciferase gene driven by the G-CSF promoter (-283/+35) (pG-CSF(-283/+35)-Luc), then were incubated with or without U0126, followed by LPS treatment for 6 h. As shown in S5A Fig, U0126 pretreatment reduced LPS-activated G-CSF promoter activity by 60%, supporting the idea that LPS-activated G-CSF promoter activity requires activation of the MEK/ERK pathway. We therefore investigated the role of ERK2 in G-CSF promoter activation and possible regulatory factors involved. Raw264.7 cells were co-transfected with the pG-CSF(-283/+35)-Luc reporter plasmid and (i) a plasmid encoding constitutively active ERK2 (CA-ERK2) or p50, Oct-2, or C/EBPβ, all of which are involved in LPS-regulated G-CSF expression, or (ii) with pairs of plasmids consisting of the C/EBPβ-encoding plasmid and the CA-ERK2, p50, or Oct-2 plasmid. As shown in Fig 3A, co-transfection with pG-CSF(-283/+35)-Luc and the plasmid encoding p50, Oct-2, C/EBPβ, or CA-ERK2 resulted, respectively, in a 0.3-, 0.7-, 6.2-, or 2.7-fold increase in luciferase activity compared to transfection with pG-CSF(-283/+35)-Luc alone. In addition, co-transfection with the pG-CSF(-283/+35)-Luc reporter plasmid, the CA-ERK2-encoding plasmid, and the plasmid encoding C/EBPβ resulted in the highest luciferase activity, with a 28-fold increase compared to the control with the Luc reporter plasmid alone. In contrast, co-transfection with the pG-CSF(-283/+35)-Luc reporter, CA-ERK2-encoding plasmid, and either the p50 or Oct-2 expression plasmid resulted in no significant increase in luciferase activity compared to that detected in the cells co-transfected with CA-ERK2-encoding plasmid alone (Fig 3A). These findings suggest the importance of the interaction between ERK2 and C/EBPβ in regulating LPS-stimulated G-CSF promoter activity. Since ERK1/2 is known to phosphorylate C/EBPβ and thereby regulate its transcriptional factor activity [26], we next examined whether LPS treatment led to ERK1/2-mediated phosphorylation of C/EBPβ. As shown in Fig 3B, LPS treatment of RAW264.7 cells for 4 h increased both the protein and phosphorylation levels of nuclear C/EBPβ, and phosphorylation level was partially inhibited, but not the protein level, by U0126 pretreatment. Serine 64 in the transactivation domain and threonine 188 in the regulatory domain of C/EBPβ, both have been proposed as potential target sites for phosphorylation by ERK [22, 27, 28]. To clarify the phosphorylation sites on C/EBPβ by LPS-activated ERK, these two amino acids were separately mutated to alanine by point mutation to create the C/EBPβ-S64A and C/EBPβ-T188A mutants. In RAW264.7 cells co-transfected with the pG-CSF(-283/+35)-Luc reporter, the CA-ERK2-encoding plasmid, and either the wild-type C/EBPβ or one of the mutant C/EBPβ constructs, a 45% decrease in luciferase activity was detected in cells with C/EBPβ T188A compared to that with wild type C/EBPβ, whereas the C/EBPβ-S64A mutant had no significant effect (Fig 3C). We then mutated the consensus C/EBPβ binding site in the G-CSF promoter to examine its role in LPS-ERK-induced G-CSF expression. Co-transfection of Raw264.7 cells with increasing amounts of the CA-ERK2-expressing plasmid (0–0.6 μg) and the pG-CSF(-283/+35)-Luc reporter carrying either the consensus C/EBPβ binding site (WT: ATTTCACAAA) or the mutant C/EBPβ binding site (GCGTCACGCA) resulted in a 2.8- to 16-fold increase in luciferase activity using reporter carrying the WT binding site, but only a 1.6- to 4.3-fold increase with the reporter construct carrying the mutant C/EBPβ binding site (Fig 3D). Taken together, these results demonstrate that the ERK2-mediated increase in G-CSF promoter activity, at least in part, involves phosphorylation of C/EBPβ at Thr188.

### U0126 inhibits LPS-induced binding of nuclear NF-κB and C/EBPβ to the G-CSF promoter region and decreases its accessibility to DNase

**Fig 4:**
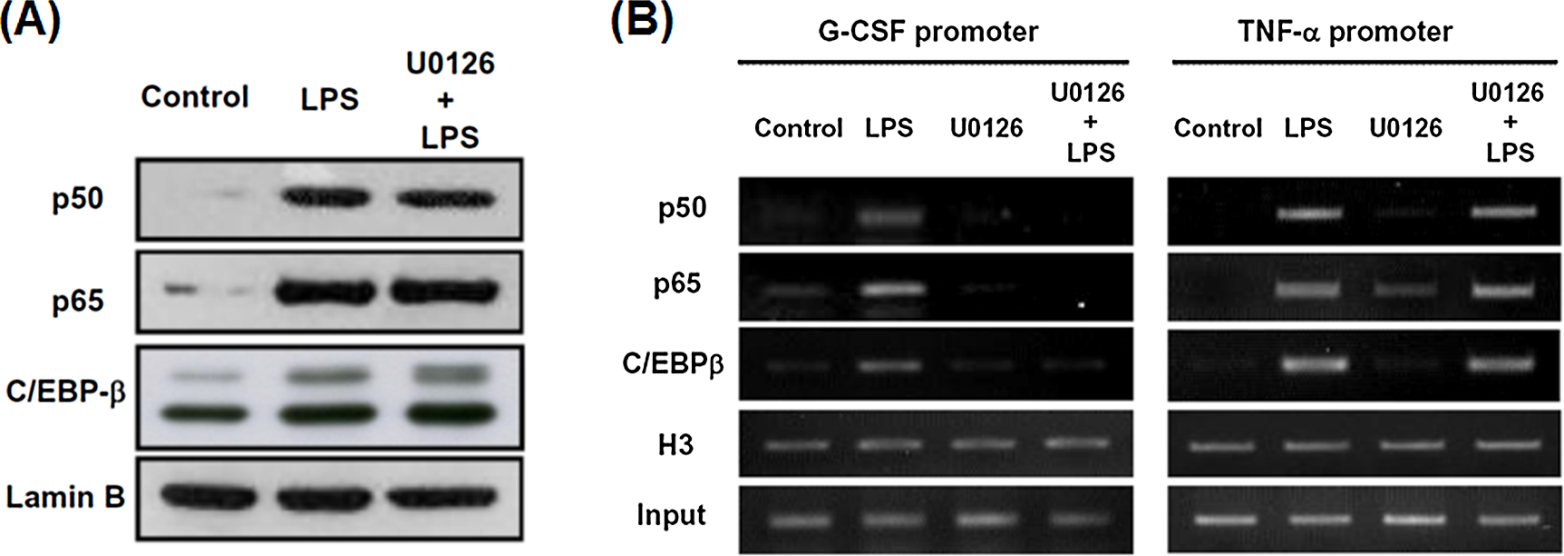
U0126 reduces LPS-induced nuclear NF-κB and C/EBPβ binding to the G-CSF promoter in Raw264.7 macrophages. Raw264.7 cells were incubated with DMSO or U0126 (10 μM) for 30 min, then with LPS (100 ng/ml) or PBS for 6 h, then the following tests were carried out. (**A**) Nuclear levels of p50, p65, C/EBPβ, and lamin B were measured by Western blotting assay. (**B**) Bindings of p50, p65, or C/EBPβ to the G-CSF promoter were analyzed by ChIP assay via precipitation with antibodies against the test protein or histone H3, used as the control, removal of the antibodies with proteinase K digestion for 12 h at 45ºC, and PCR amplification of a 179-bp G-CSF promoter fragment (-248 to -70 bp) or a 274 bp TNF-α promoter fragment (-270 to -4 bp). Ten per cent of the chromatin DNA used for immunoprecipitation was subjected to PCR and is indicated as ‘input’ (bottom row). In A and B, the data are representative of the results from three independent experiments.

To determine whether U0126 inhibited LPS-induced G-CSF expression by affecting transcription factors known to regulate G-CSF expression, we analyzed levels of the transcriptional regulators including p50, p65, and C/EBPβ in the nucleus of RAW264.7 cells. As shown in Fig 4A, nuclear levels of p50, p65, and C/EBPβ were increased after 6 h of LPS treatment and this effect was unaffected by pretreatment with U0126. These results show that nuclear levels of these transcriptional factors are irrelevant for the ERK-regulated G-CSF transcription. We then used ChIP assays to investigate the effect of U0126 on the bindings of p50, p65, and C/EBPβ to the promoters of G-CSF and TNF-α in cells treated with LPS. Fig 4B shows that binding of p50, p65, or C/EBPβ to the G-CSF promoter and the TNF-α promoter was induced by LPS and that U0126 pretreatment inhibited binding of all three to the G-CSF promoter, but not the TNF-α promoter (Fig 4B). To clarify whether U0126 pretreatment affected LPS-stimulated NF-κB transcriptional activity, Raw264.7 cells were transfected with an NF-κB-driven luciferase reporter (pNF-κB-Neo-Luc), then, at 24 h after transfection, were incubated with LPS with or without U0126 pretreatment. As expected, LPS treatment for 6 h significantly upregulated NF-κB-driven luciferase activity and this effect was not affected by U0126 pretreatment (S5B Fig). These results indicate that LPS-triggered NF-κB activation, including nuclear levels of NF-κB and its transactivation activity, was not affected by U0126.

**Fig 5:**
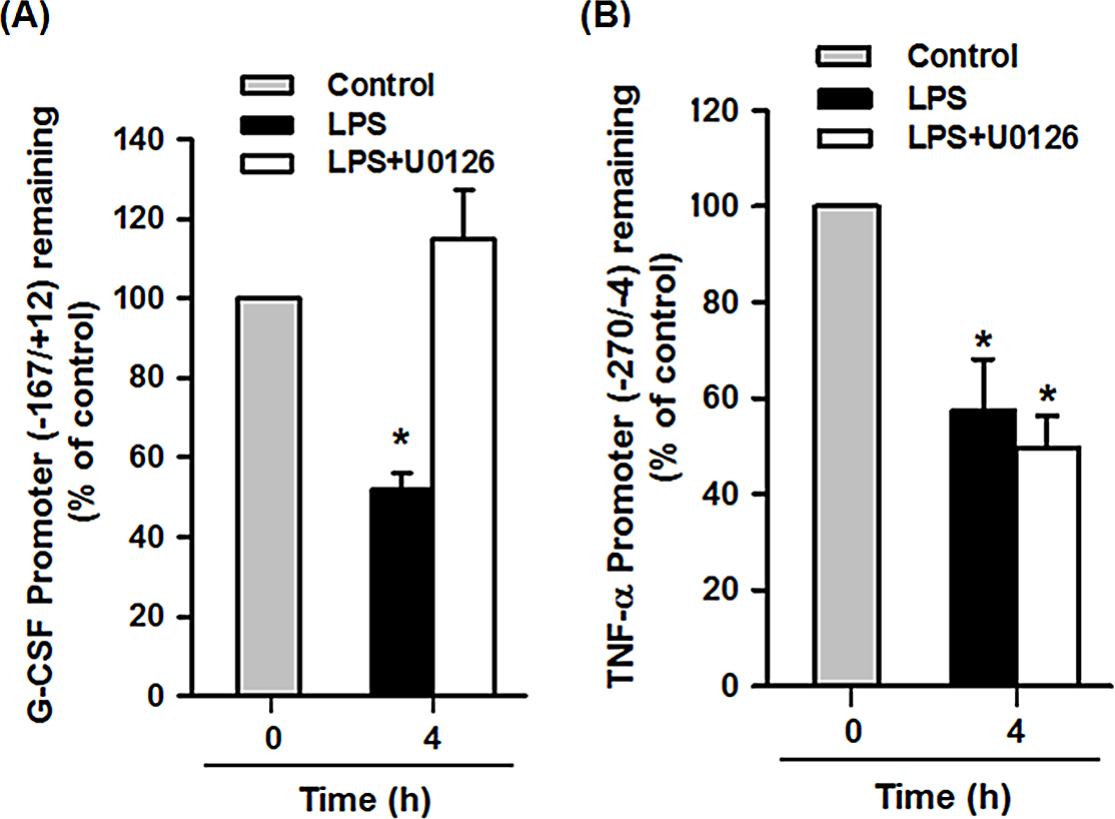
U0126 decreases the LPS-induced increased accessibility of the G-CSF promoter to DNase I. Raw264.7 macrophages were pretreated with DMSO or U0126 (10 μM), then incubated with LPS (100 ng/ml) or PBS for 4 h. Nuclei were then isolated and subjected to DNase I digestion for 2 min, then genomic DNA was isolated and quantitative real-time PCR was performed to measure the amount of undigested DNA in region -167/+12 of the G-CSF promoter (**A**) and in region -270/-4 of the TNF-α promoter (**B**), which is expressed as a percentage of that in the control cells. The results are the mean ± SD for three independent experiments. *p<0.05 compared to the control.

**Fig 6:**
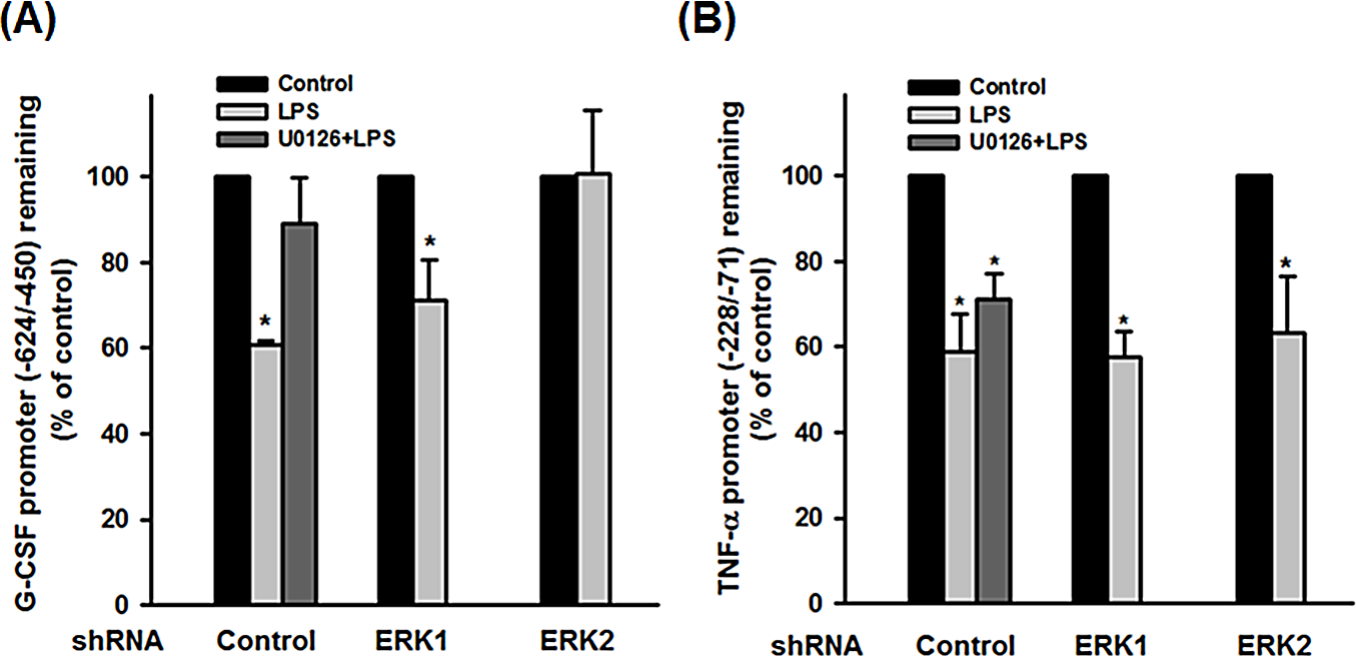
Knockdown of ERK2, but not ERK1, decreases LPS-induced DNase I accessibility of the G-CSF promoter. THP-1 cells in which either ERK1 or ERK2 was knocked down using shRNA were induced to differentiate into macrophages. After incubation with DMSO or U0126 (10 μM), the cells were incubated with LPS (100 ng/ml) or PBS for 4 h, then nuclei were purified and subjected to DNase I digestion for 5 min. Genomic DNA was then isolated and the amount of undigested DNA within region -624/-450 of the G-CSF promoter (**A**) or within region -228/-71 of the TNF- α promoter (**B**) was determined by quantitative real-time PCR and expressed as a percentage of that in cells not treated with LPS and U0126. The results are the mean ± SD for three independent experiments. *p<0.05 compared to the corresponding control.

Activation of the MEK-ERK signaling pathway induces histone phosphorylation, causing nucleosome remodeling at the promoters of immediate early genes [29]. DNase accessibility analysis was therefore employed to determine whether LPS activation of ERK resulted in modulation of chromatin remodeling at the promoter region of the G-CSF gene. Nuclei of RAW264.7 or THP-1 macrophages pretreated with DMSO or U0126, then treated with LPS for 4 h were subjected to DNase I digestion and the amount of undigested DNA in the G-CSF and TNF-α promoter regions containing C/EBPβ binding sites was determined by quantitative real-time PCR. In Raw264.7 cells, the amount of undigested DNA in region -167/+12 of the G-CSF promoter (Fig 5A) and region -270/-4 of the TNF-α promoter (Fig 5B) was decreased after LPS treatment, while U0126 pretreatment restored the amount of undigested G-CSF promoter DNA, but not TNF-α promoter DNA, to the control level. Similar results were detected in human THP-1 macrophages that LPS treatment decreased the amounts of undigested DNA within the promoter regions of G-CSF (Fig 6A) and TNF-α (Fig 6B) that contain a C/EBPβ binding site. Again, pretreatment with U0126 restored the amount of undigested DNA within the G-CSF promoter, but not the TNF-α promoter (Fig 6A and 6B). Furthermore, knockdown of ERK2 in THP-1 cells resulted in an increased amount of undigested G-CSF promoter DNA (Fig 6A), but had no significant effect on the TNF-α promoter (Fig 6B) in LPS-treated cells compared to LPS-treated knockdown control cells. Whereas, ERK1 knockdown had no significant effect on the amount of undigested G-CSF and TNF-α promoter DNA in LPS-treated cells compared to LPS-treated control knockdown cells. These results reveal that LPS treatment increases the accessibility of both the G-CSF and TNF-α promoters to DNase I and that U0126 pretreatment or ERK2 depletion abrogates LPS effect on DNase accessibility of the G-CSF promoter, but not the TNF-α promoter region.

## Discussion

Although recombinant G-CSF is widely used clinically, regulation of endogenous G-CSF expression has not been well characterized. In this study, we used a highly selective inhibitor of MAP/ERK kinase, U0126, and shRNA knockdown of ERK1 or ERK2 to demonstrate that LPS-induced activation of ERK2, but not ERK1, was required for LPS-induced G-CSF expression in macrophages. Moreover, our results showed that LPS-activated ERK2 interacts with C/EBPβ and increases the accessibility of the G-CSF promoter to DNase I and transcriptional factor binding to the G-CSF promoter, leading to up-regulation of G-CSF expression.

ERK signaling is known associated with various cellular processes, including proliferation, differentiation, and survival. The two classical ERKs, ERK1 and ERK2, co-express in most tissues, but vary in relative abundance. They involve in the transcriptional regulation of multiple cellular processes by phosphorylating many substrates, including Elk1, c-Myc, and ribosomal S6 kinase, and by activating c-fos. An animal study showed that ERK2 knockout is lethal and cannot be compensated for by ERK1, while ERK1 knockout has only a mild phenotypic effect [30]. It is therefore proposed that ERK1 and ERK2 have different biological functions [31]. It is also known that ERK2 expression is more critical than ERK1 expression for survival [31]. In this study, we evidenced that ERK2, rather than ERK1, involved in LPS-induced G-CSF expression in macrophages, further illustrating the different functions of these two kinases.

ERK2 has been identified as a transcription regulator but the consequence of ERK2 signaling might be determined by the interaction of ERK2 and other proteins in either the nucleus or cytoplasm, such as nuclear factors, scaffold proteins, and anchoring proteins. For example, interaction between activated ERK2 and the IκB-NF-κB complex in the cytoplasm has been implicated in protecting myeloid leukemia cells against apoptogenic stimuli [32]. Although NF-κB is known to be a major transcriptional regulator of the expressions of inflammatory genes, NF-κB alone is not sufficient to drive transcription of G-CSF [4]. We have previously shown that, following LPS stimulation, Oct-2 binds to the promoters of the iNOS and G-CSF genes and this effect is reduced by inhibitors of PI3K, AKT, or mTOR, thus demonstrating the essential role of Oct-2 in regulating the transcription of these genes [6]. All these results suggest that both Oct-2 and NF-κB are essential for LPS-induced expression of G-CSF. However, as shown in Fig 4 and S6 Fig in this study, U0126 did not affect LPS-induced nuclear levels of NF-κB (p50 and p65) and Oct-2, but reduced the LPS-induced binding of NF-κB p50 and p65 to the G-CSF promoter, but not to the TNF-α promoter. In addition, LPS-induced accessibility of the G-CSF promoter to DNase I was prevented by U0126 or ERK2 knockdown, but not by ERK1 knockdown, and, again, no such effect was seen with the TNF-α promoter region (Figs 5 and 6). These results further suggest that ERK2 activation may specifically alter chromatin conformation in the G-CSF promoter region, thus increasing its accessibility to DNase I digestion and facilitating the binding of transcription factors [33]. Carlson et al. [34] identified a potential ERK2 substrate, ETV3, a transcriptional regulator that is extensively phosphorylated by activated ERK2 and loses its ability to bind to the GGAA-containing E box motif at the promoters of many genes. ETV3 has been suggested to function by cooperating with SBNO2, a transcriptional corepressor, to regulate the anti-inflammatory effects of IL-10 [35]. In our study here, we provide evidences that ERK2 mediates LPS-upregulated G-CSF promoter activity, at least in part, by interacting with C/EBPβ and increasing its ability to activate transcription. However, it remains unclear whether factors similar to ETV3 or other chromatin remodeling complexes involve in LPS-ERK2-regulated G-CSF expression in macrophages.

NF-κB controls many genes involved in inflammation, such as G-CSF, IL-6, TNF-α and iNOS. Although we show that U0126 has no effect on LPS-increased NF-κB-dependent reporter gene expression and nuclear protein levels of p50 and p65 in RAW 264.7 macrophage. LPS-induced NF-κB activation has been reported to be inhibited by PD98059 in murine J774 macrophages [36]. The discrepancy could be due to that different cells or different methods were used. In addition, studies have shown that inhibition of ERK1/2 has no effect on the activation of a stably transfected NF-κB-dependent reporter gene expression [37], or expression of NF-κB down-stream genes, such as IL-6, TNF-α and iNOS [38–40]. Furthermore, evidence showing that phosphorylation level of p65 at S468 and S536 is not inhibited by PD98059 [41, 42]. These results suggest that ERK2 may not a critical regulator of NF-κB activation, and ERK2 regulates G-CSF expression is not through inhibition of NF-κB activation.

G-CSF is essential for the protective inflammatory response and for maintaining the balance between anti- and pro-inflammatory reactions in the inflammatory condition. Higher levels of G-CSF may cause an excessive inflammatory response and have been associated with morbidity and mortality of acute lung injury or with chronic inflammatory diseases, such as rheumatoid arthritis [9, 10]. Knockout of G-CSF protects mice against collagen-induced arthritis, mostly due to prevention of infiltration by activated neutrophils [43]. Thus, inhibition of G-CSF activity is now considered as a new therapeutic strategy for rheumatoid arthritis and other inflammatory diseases.

In this report, we provide evidences for a pivotal role of ERK2 in LPS-induced G-CSF expression in macrophages. We show that ERK2 involves in LPS-induced G-CSF expression at two distinct steps; activation of ERK2 triggers local chromatin remodeling, which may facilitate binding of transcription factors to the G-CSF promoter, and activation of ERK2 cooperates with C/EBPβ to activate G-CSF promoter activity. These effects of ERK2 on G-CSF expression are not shared by ERK1. The precise mechanism by which ERK2 causes chromatin remodeling is not known yet and further studies are needed.

## Supporting Information

S1 Fig Effects of inhibitors of the PI3K, MAPK, or PKC pathways on LPS-induced G-CSF protein in culture medium of RAW264.7 macrophages. RAW264.7 cells were pretreated for 30 min with DMSO, a PI3K inhibitor (50 μM LY294002), a MEK inhibitor (10 μM U0126), a JNK inhibitor (0.5 μM L-JNKi 1 trifluoroacetate), a p38 inhibitor (20 μM SB203580), or a PKC inhibitor (1 μM RO318220), then 100 ng/ml of LPS was added for 6 h, then levels of G-CSF protein in the culture medium were measured by ELISA. The values are the mean ± SD for three separate experiments. *p < 0.01 compared to the DMSO-treated cells.
(TIF)

S2 Fig U0126 or PD98059 inhibits LPS-induced increase of G-CSF mRNA in bone marrow-derived macrophages. Mouse bone marrow-derived macrophages (BMDMs), cultured as described previously [Arch Biochem Biophys. 2011;508: 110–119.], were left untreated (lane 1) or were pretreated with (**A**) DMSO or 0.01 or 0.1 μM U0126 (lanes 2–5) or (**B**) DMSO or 1 or 10 μM PD98059 (lanes 2–5), then were incubated with LPS (100 ng/ml) or PBS for 6 h. Total RNA was then isolated and the levels of G-CSF and GAPDH (internal control) mRNA were determined by RT-PCR and analyzed by gel electrophoresis. The data shown are typical of the results obtained in two independent experiments.
(TIF)

S3 Fig U0126 inhibits ERK1/2 phosphorylation in LPS-stimulated THP-1 macrophages. PMA differentiated THP-1 macrophages were left untreated (lane 1) or were incubated either with LPS (100 ng/ml) for 0.5 to 2 h (lanes 2–4) or pretreated with U0126 (10 μM) for 30 min, followed by addition of same concentration of LPS and incubation for 2 h (lane 5), then phosphorylated ERK1/2 and total ERK1/2 were analyzed by Western blotting.
(TIF)

S4 Fig Knockdown of ERK1 and ERK2 by shRNA. THP-1 cells were infected with lentivirus carrying specific shRNAs for ERK1 (ERK1a and ERK1b) or ERK2 (ERK2a and ERK2b) and selected with puromycin (10 μg/ml) for 10 days, then the levels of ERK1/2 and β-actin in the cells were determined by Western blotting.
(TIF)

S5 Fig U0126 inhibits LPS-induced G-CSF promoter but not NF-κB-driven promoter activity. RAW264.7 cells were co-transfected with 1 μg of pG-CSF(−283/+35)-Luc (**A**) or the pTransNF-κB-Neo plasmid (**B**) and 0.05 μg of phRLTK. At 24 h post-transfection, the cells were pretreated with DMSO or 10 μM U0126 for 30 min, followed by addition of LPS (100 ng/ml) for 6 h, then luciferase activities were determined using the Dual-Luciferase reporter assay system, and firefly luciferase activity was normalized to renilla luciferase activity, then the results were expressed relative to those for untreated control cells (C). The values are the mean ± SD for three independent experiments. *p < 0.01 compared to the LPS-treated cells in (**A**) and *p < 0.05 compared to the LPS-treated cells in (**B**).
(TIF)

S6 Fig Effect of U0126 on the LPS-induced increase in nuclear Oct-2 protein. Raw264.7 cells were left untreated (lane 1) or were treated with LPS (100 ng/ml) for 6 h (lane 2) or were pretreated with U0126 (10 μM) for 30 min, then treated with LPS (100 ng/ml) for 6 h (lane 3), then nuclei were isolated and nuclear levels of Oct-2 and lamin B were determined by Western blotting.
(TIF)

S1 Table Sequences of the oligonucleotides used for RT-PCR. (DOCX)
