# Supplementary figures and images for: LPS-Induced G-CSF Expression in Macrophages Is Mediated by ERK2, but Not ERK1
Source: PLoS One. 2015 Jun 26;10(6):e0129685. doi: 10.1371/journal.pone.0129685 (PMC4483241; doi:10.1371/journal.pone.0129685)

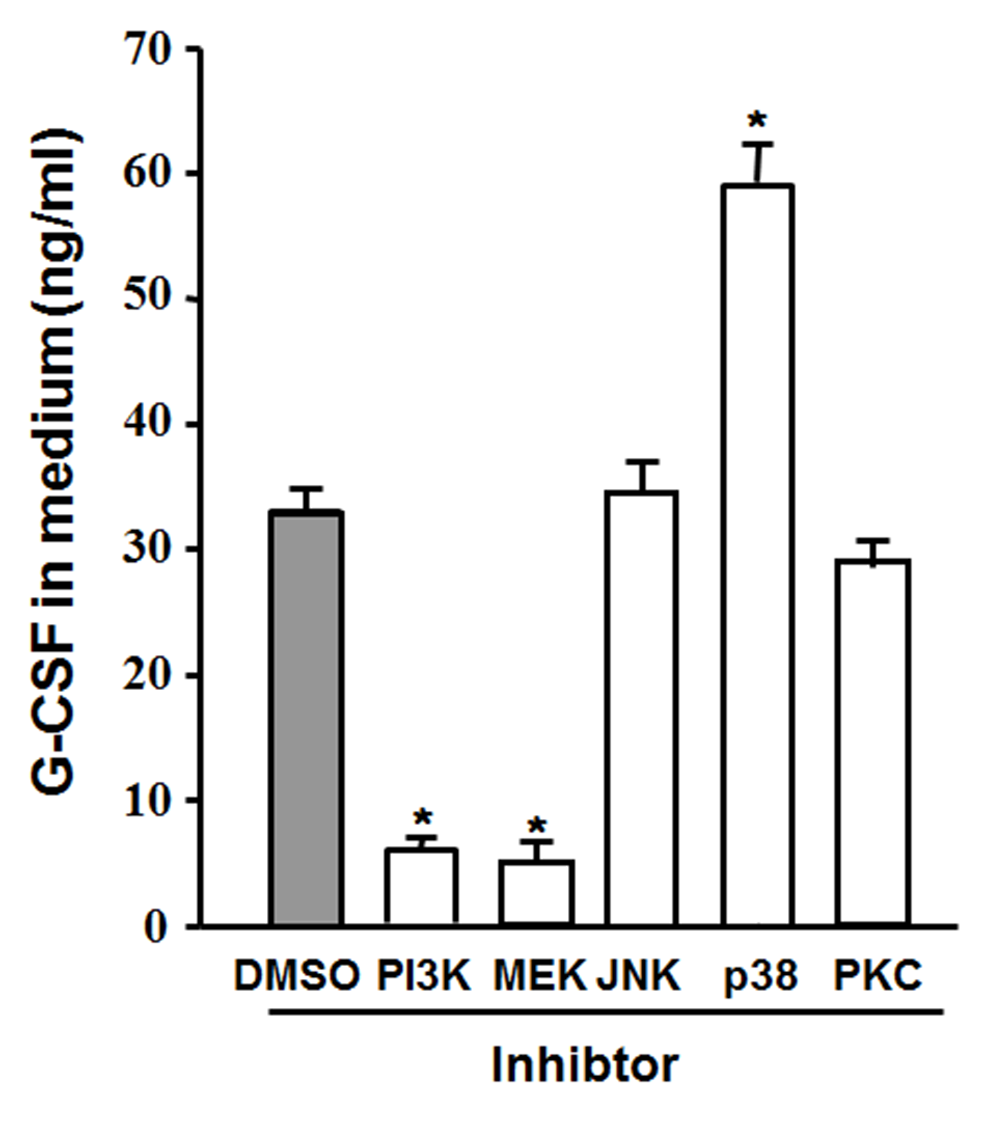

Supplement: S1 Fig — RAW264.7 cells were pretreated for 30 min with DMSO, a PI3K inhibitor (50 μM LY294002), a MEK inhibitor (10 μM U0126), a JNK inhibitor (0.5 μM L-JNKi 1 trifluoroacetate), a p38 inhibitor (20 μM SB203580), or a PKC inhibitor (1 μM RO318220), then 100 ng/ml of LPS was added for 6 h, then levels of G-CSF protein in the culture medium were measured by ELISA. The values are the mean ± SD for three separate experiments. *p < 0.01 compared to the DMSO-treated cells. (TIF) [file pone.0129685.s001.tif]

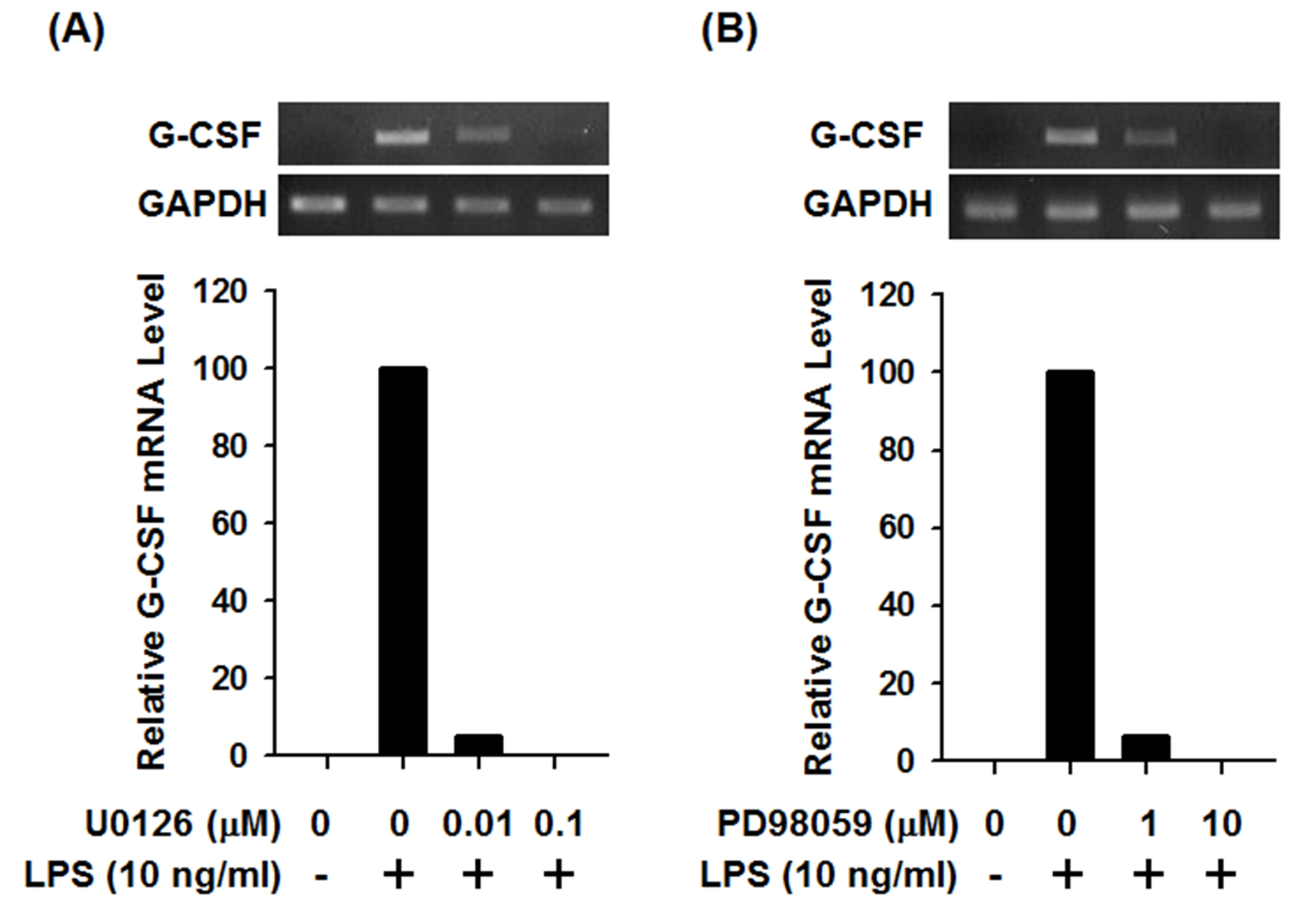

Supplement: S2 Fig — Mouse bone marrow-derived macrophages (BMDMs), cultured as described previously [Arch Biochem Biophys. 2011;508: 110–119.], were left untreated (lane 1) or were pretreated with (A) DMSO or 0.01 or 0.1 μM U0126 (lanes 2–5) or (B) DMSO or 1 or 10 μM PD98059 (lanes 2–5), then were incubated with LPS (100 ng/ml) or PBS for 6 h. Total RNA was then isolated and the levels of G-CSF and GAPDH (internal control) mRNA were determined by RT-PCR and analyzed by gel electrophoresis. The data shown are typical of the results obtained in two independent experiments. (TIF) [file pone.0129685.s002.tif]

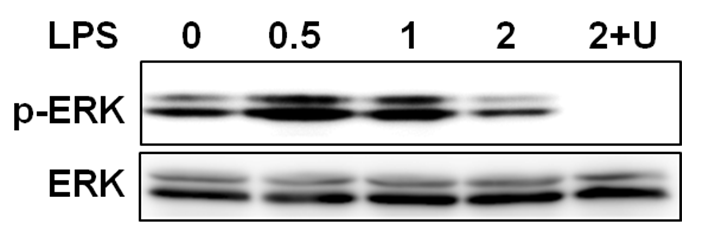

Supplement: S3 Fig — PMA differentiated THP-1 macrophages were left untreated (lane 1) or were incubated either with LPS (100 ng/ml) for 0.5 to 2 h (lanes 2–4) or pretreated with U0126 (10 μM) for 30 min, followed by addition of same concentration of LPS and incubation for 2 h (lane 5), then phosphorylated ERK1/2 and total ERK1/2 were analyzed by Western blotting. (TIF) [file pone.0129685.s003.tif]

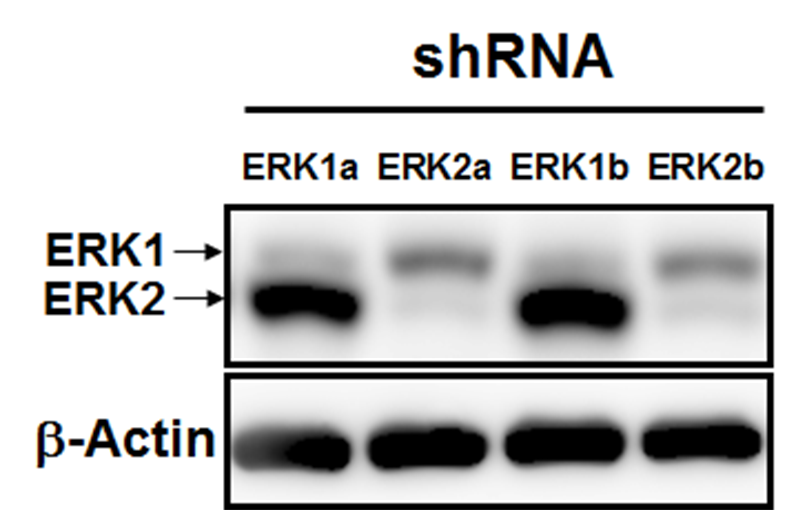

Supplement: S4 Fig — THP-1 cells were infected with lentivirus carrying specific shRNAs for ERK1 (ERK1a and ERK1b) or ERK2 (ERK2a and ERK2b) and selected with puromycin (10 μg/ml) for 10 days, then the levels of ERK1/2 and β-actin in the cells were determined by Western blotting. (TIF) [file pone.0129685.s004.tif]

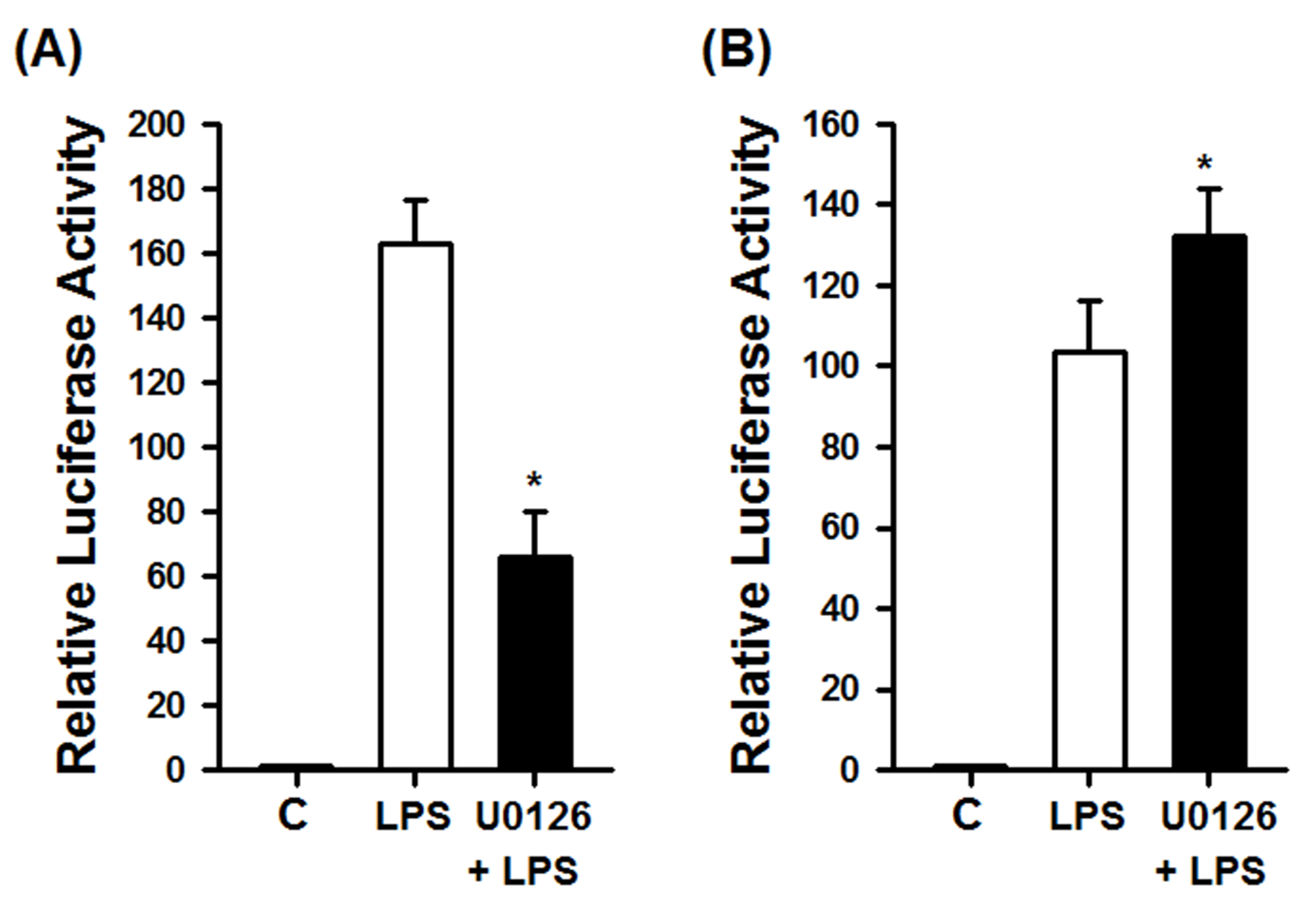

Supplement: S5 Fig — RAW264.7 cells were co-transfected with 1 μg of pG-CSF(−283/+35)-Luc (A) or the pTransNF-κB-Neo plasmid (B) and 0.05 μg of phRLTK. At 24 h post-transfection, the cells were pretreated with DMSO or 10 μM U0126 for 30 min, followed by addition of LPS (100 ng/ml) for 6 h, then luciferase activities were determined using the Dual-Luciferase reporter assay system, and firefly luciferase activity was normalized to renilla luciferase activity, then the results were expressed relative to those for untreated control cells (C). The values are the mean ± SD for three independent experiments. *p < 0.01 compared to the LPS-treated cells in (A) and *p < 0.05 compared to the LPS-treated cells in (B). (TIF) [file pone.0129685.s005.tif]

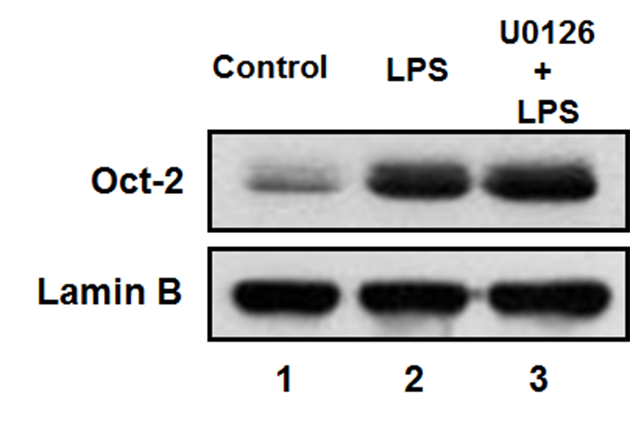

Supplement: S6 Fig — Raw264.7 cells were left untreated (lane 1) or were treated with LPS (100 ng/ml) for 6 h (lane 2) or were pretreated with U0126 (10 μM) for 30 min, then treated with LPS (100 ng/ml) for 6 h (lane 3), then nuclei were isolated and nuclear levels of Oct-2 and lamin B were determined by Western blotting. (TIF) [file pone.0129685.s006.tif]
