# Supplementary material for: LPS-Induced G-CSF Expression in Macrophages Is Mediated by ERK2, but Not ERK1
Source: PLoS One. 2015 Jun 26;10(6):e0129685. doi: 10.1371/journal.pone.0129685 (PMC4483241; doi:10.1371/journal.pone.0129685)
Supplement: S1 Table — (DOCX) [file pone.0129685.s007.docx]

**S1 Table. Sequences of the oligonucleotides used for RT-PCR.**

| Primers for PCR | Sequence |
| --- | --- |
| TNF-α | Forward 5’-AATGGACCCGACATTAACCA-3’ |
|  | Reverse 5’-AAATGGTCGTTTGGCTGAAG-3’ |
| G-CSF | Forward 5’-CTCAACTTTCTGCCCAGAGG-3’ |
|  | Reverse 5’-CTGGAAGGCAGAAGTGAAGG-3’ |
| GAPDH | Forward 5’-AAAGGATCCACTGGCGTCTTCACCACC-3’ |
|  | Reverse 5’-GAATTCGTCATGGATGACCTTGGCCAG-3’ |
| Primers for DNase I accessibility assay | Sequence |
| mG-CSF | Forward 5’-TGGCTGGAAGAGAGGAAGAG-3’ |
|  | Reverse 5’-CTGGGGCAACTCAGGCTTA-3’ |
| mTNF-α | Forward 5’-CTGATTGGCCCCAGATTG-3’ |
|  | Reverse 5’-CTTCTGCTGGCTGGCTGT-3’ |
| hG-CSF | Forward 5’-CGGGATTCTAGGGGCTTTAG-3’ |
|  | Reverse 5’-CCTGAGCCCCTACCTGAGA-3’ |
| hTNF-α | Forward 5’-GGGAGTGTGAGGGGTATCCT-3’ |
|  | Reverse 5’-CAACCAGCGGAAAACTTCCTT-3’ |

h: human; m, mouse.
